# Bilateral Canal of Nuck Cysts Presenting as Bartholin′s Cysts: A Case Report

**DOI:** 10.1155/crog/5816734

**Published:** 2026-09-16

**Authors:** Reem Chamseddine, Yevgeniya Osipova

**Affiliations:** ^1^ The Brooklyn Hospital Center, Brooklyn, New York, USA, tbh.org; ^2^ Advantage Care Physicians, Brooklyn, New York, USA

**Keywords:** Bartholin′s gland, canal of Nuck, case report

## Abstract

We present the case of a 24‐year‐old woman with a presentation consistent with Bartholin′s cysts ultimately identified as cysts originating from the ducts of the canal. Our study of this patient underscores the need for a comprehensive diagnostic approach in recurrent vulvar cysts. This case illustrates how overlapping clinical features can obscure less common conditions, leading to potential misdiagnosis. Our study of this patient emphasizes the importance of maintaining a broad differential diagnosis when evaluating recurrent vulvar cysts when patients do not respond to standard treatment approaches. Although Bartholin′s gland cysts are commonly encountered, canal of Nuck cysts require careful differentiation, through a pathology‐based diagnosis, and definitive management through surgery alone.

## 1. Introduction

Young female patients commonly present to the clinic or the emergency department with vulvar symptoms. The differential diagnosis for vulvar swelling is wide, ranging from benign masses (e.g., lipomas or angiofibroblastomas) to malignant tumors (e.g., squamous cell carcinoma or leiomyosarcoma). [1] Among the benign conditions, Bartholin′s gland cysts are perhaps the most common, affecting an estimated 2% of women during their lifetime. [2] Bartholin′s cysts do not typically require radiographic or laboratory evaluation; however, a pathology‐based diagnosis may be warranted even in the setting of typically presenting Bartholin′s cysts. This case report describes a 24‐year‐old woman with recurrent vulvar cysts that were eventually diagnosed as originating from the canal of Nuck.

## 2. Case Presentation

**Figure 1:**
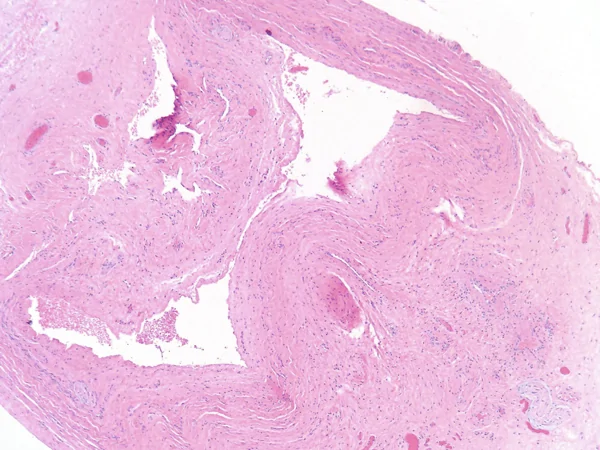
Pathology slide showing from specimen showing flat, morphologically mesothelial cells with no vascularization.

A 24‐year‐old nulligravid woman presented to the clinic with a painful swelling in the right labium minus. She had no history of local trauma and no symptoms suggestive of infection. Her body mass index was 20. Her medical history was negative, as was her surgical history. Her physical examination was notable for what appeared to be bilateral Bartholin′s cysts at the 4 and 8 o′clock positions of the vaginal introitus. Thus, the patient underwent a bilateral incision and drainage (I&D) procedure in the office. The patient returned to the clinic 2 weeks later complaining of recurrent swelling. The decision was made to proceed with a repeat I&D and Word catheter placement bilaterally. The patient returned to the clinic 6 weeks later, reporting, for the second time, recurrence of swelling bilaterally. At this time, the patient underwent a marsupialization procedure, which again did not prevent recurrence. After counseling, the patient agreed to surgical management. Seven months after initial presentation, the patient underwent an excision of the Bartholin′s cysts bilaterally in the operating room. Postoperatively, the patient developed a large, right‐sided vulvar hematoma that resolved after surgical evacuation. The results of pathological diagnosis for the cyst wall and contents returned as “benign cysts lined by mesothelial cells, suggestive of cysts of canal of Nuck”(Figure 1). At her postoperative visit, the patient was doing well with well‐healing surgical sites and no evidence to suggest recurrence.

**Figure 2:**
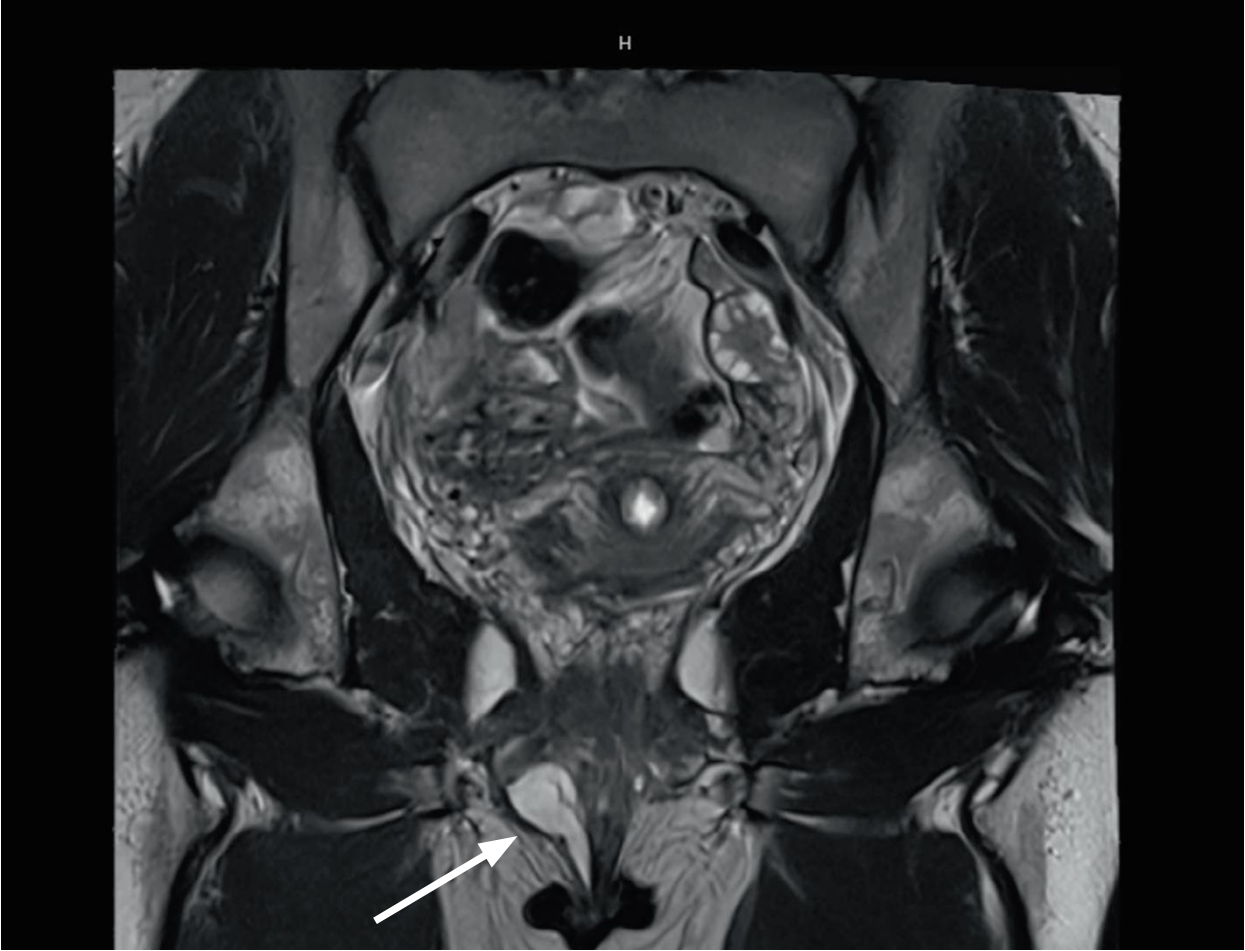
Canal of Nuck cyst as seen on MRI, incorrectly characterized as Bartholin′s cyst abscess.

Three months later, the patient had recurrence on one side. At that time, the patient had undergone magnetic resonance imaging (MRI) of the pelvis, which revealed a right‐sided Bartholin′s cyst (Figure 2). At the time of writing, the patient had elected to proceed with expectant management and no further surgical intervention was attempted.

## 3. Discussion

Cysts of the canal of Nuck can present a diagnostic challenge due to their rarity and similarity to the much more commonly occurring Bartholin′s gland cysts.

There are no published studies about the incidence rates of canal of Nuck cysts in adult women. A review of the literature as of August 2024 resulted in fewer than 30 case reports detailing unique presentations among adult and pediatric female patients, with a single case report about a bilateral presentation such as the one seen in our patient. [3]

The canal of Nuck is named after anatomist Anton Nuck, who first described this potential space in the late 17th century. [4] This canal is a remnant of the processus vaginalis, which typically closes during fetal development or within the first year of life. [5] The failure of the processus vaginalis to obliterate results in persistence of parietal peritoneum through the inguinal canal to the labia majora. [6] When the canal fills with fluid, the female patient develops a cyst, also referred to as a hydrocele in the literature. Based on the degree of patency of the canal, cysts may present as inguinal masses rather than vulvar masses. [7] In some cases, the canal of Nuck masses contain herniated contents and present as hernias rather than cysts. [8]

With regards to diagnosis, ultrasound and MRI are commonly cited as possible imaging modalities in the literature, with ultrasound showing anechoic or hypoechoic well‐defined masses in the areas of a canal of Nuck cysts. [9] In MRI, the cyst appears hypointense on T1‐weighted images and hyperintense on T2‐weighted images. Internal septations within the cysts can be seen on ultrasonography and MRI. [10] With regards to management, surgery appears to be the definitive treatment option, although no procedure has been identified as the standard of care. Cited approaches include simple cystectomy (as in the case of our patient), inguinal ring ligation, and round ligament excision. [11]

This patient presentation highlights the importance of pathology‐based diagnosis in the setting of recurrent vulvar cysts. Although canal of Nuck cysts are lined by a single layer of mesothelial cells, Bartholin′s gland cysts are typically lined by mucinous epithelium or transitional epithelium. This subtle difference, only detectable through study of the tissue, is crucial for correct diagnosis and subsequent appropriate surgical management. The existing literature supports the importance of making the correct diagnosis due to the range of phenotypes in the community (Table 1). In the case of our patient, even advanced imaging with MRI was not sufficient for diagnosis. The rarity of this clinical entity poses a challenge to practitioners familiar with the much more commonly occurring Bartholin′s cysts.

**Table 1:** Canal of Nuck cysts in literature: case reports 2020–2025.

Cases in literature	Initial presentation	Suspected diagnosis and unique finding
Kohlhauser et al. [12]	Painful right groin mass	Inguinal hernia
Shahid et al. [13]	Painful right groin swelling	Inguinal hernia
Wang et al. [14]	Painful left groin swelling	Inguinal hernia
Baig et al. [15]	Painful right groin lump	Femoral hernia
Fukahori et al. [16]	Painless right inguinal mass	Inguinal hernia; concurrent finding of femoral hernia with ruptured abdominal canal of Nuck cyst
Bumann and Corra [17]	Painful left mass with cyclical change in size	Irritable bowel syndrome and Endometriosis were differential diagnoses
Hwang et al. [18]	Painful left groin mass	Concurrent endometriosis inside the cyst
Chudasama et al. [19]	Painless left inguinal swelling	Elderly patient at 85 years of age
Prevezanos et al. [20]	Painful right inguinal mass	Incarcerated inguinal hernia
Hang et al. [21]	Groin mass with menstrual cycle‐related pain	Inguinal hernia
Mete Ural et al. [22]	Swelling of left inguinal region	Presence of endometriosis‐like lesions inside the cyst
Meskel et al. [23]	Painless right inguinal swelling	Benign cyst; Pediatric patient at 6 years of age
Long et al. [24]	Painless left inguinal mass with menstrual cycle–related change in size	N/A; use of robotic‐assisted laparoscopic surgery

## Funding

No funding was received for this manuscript.

## Consent

A written consent form was signed by the patient.

## Conflicts of Interest

The authors declare no conflicts of interest.

## Data Availability

The data that support the findings of this study are available on request from the corresponding author. The data are not publicly available due to privacy or ethical restrictions.
